# A qualitative study on behavioral and social drivers of COVID-19 vaccine amongst refugees and migrants in Pakistan

**DOI:** 10.1371/journal.pgph.0004444

**Published:** 2025-04-08

**Authors:** Zahra Ali Padhani, Maryam Hameed Khan, Rahima Yasin, Abdu R. Rahman, Sohail Lakhani, Mushtaq Mirani, Muhammad Khan Jamali, Zahid Ali Khan, Sana Khatoon, Riya Partab, Atta ul Haq, Vinay Kampalath, Seyed-Moeen Hosseinalipour, Karl Blanchet, Jai K. Das

**Affiliations:** 1 Institute for Global Health and Development, Aga Khan University, Karachi, Pakistan; 2 School of Public Health, Faculty of Health and Medical Sciences, University of Adelaide, Adelaide, South Australia, Australia; 3 Robinson Research Institute, Faculty of Health and Medical Sciences, University of Adelaide, Adelaide, South Australia, Australia; 4 Youth Association for Development, Quetta, Pakistan; 5 University of Pennsylvania, Philadelphia, Pennsylvania, United States of America; 6 Geneva Centre of Humanitarian Studies, Faculty of Medicine, University of Geneva, Geneva, Switzerland; 7 Departmen t of Paediatrics and Child Health, Aga Khan University, Karachi, Pakistan; University of Washington Seattle Campus: University of Washington, UNITED STATES OF AMERICA

## Abstract

Migrants and refugees are among the most disadvantaged populations, with limited evidence on the access and uptake of COVID-19 vaccination among them. Therefore this qualitative study explores the behavioral and social drivers of the COVID-19 vaccine among the refugee and migrant population in Pakistan through in-depth interviews and focus group discussions with regular and irregular migrants and refugees residing in Pakistan. Key informant interviews were conducted with stakeholders responsible for overlooking the COVID-19 vaccination process. A total of 18 participants were interviewed to gather insights on COVID-19 vaccine access, uptake, and behaviours among migrants and refugees. Data was collection from June to July 2022, in Karachi, Hyderabad, and Quetta. All the interviews were audio recorded, transcribed, translated, and thematically analysed on Nvivo software. The study found that refugee and migrant communities in Pakistan faced significant challenges to COVID-19 vaccination uptake, with barriers including misconceptions about vaccine safety and efficacy, fears of side effects, and mistrust spread by religious leaders. Participants were refused vaccinations at many centers despite government directives allowing vaccines for those without Computerized National Identity Cards (CNIC). Limited outreach and awareness efforts from the government, fears of identification and deportation, long wait times at vaccination centers, and the absence of female vaccinators in communities with strict gender norms further hindered access. Many participants also reported being charged for vaccination leading to lower vaccine coverage. Despite these challenges, some individuals were motivated to vaccinate due to workplace requirements, peer influence, or personal health concerns. Facilitators included door-to-door vaccination campaigns and school vaccination mandates. Vaccination camps set up by NGOs and government agencies at border areas and migrant-rich districts facilitated access. The study suggests targeted strategies to improve vaccination coverage, including provision of identification documents to migrants, inclusion in policy, and enforcement of multilingual communication to improve healthcare access.

## Introduction

Pakistan is a resource-limited country, allocating only 2.95% of its GDP to healthcare, in contrast to the average expenditure of 8.69% in other high income countries [1]. Due to its proximity to Afghanistan, China, Iran, and India, Pakistan receives a significant influx of immigrants, primarily from Afghanistan, a country marked by considerable political instability [2]. Currently, Pakistan hosts a total of 2.2 million population of refugees and migrants from Afghanistan, Bangladesh, Myanmar, Somalia, Syria, and Yemen [2–5]. The major challenges faced by these populations include access to necessities such as education, housing, jobs, and healthcare [2].

As a low- and middle-income country (LMIC), Pakistan faced significant concerns regarding its capacity to manage a full-scale pandemic effectively. These challenges were further compounded by its geographic proximity to high-risk regions. On February 26, 2020, Pakistan reported its first case of COVID-19 in Karachi, one of its biggest metropolitan cities with a population of 16 million people [6, 7]. The virus initially entered Pakistan through returning pilgrims from Iran and Saudi Arabia, as well as Pakistani citizens stranded in other countries who were repatriated on special flights [8, 9]. In the wake of this outbreak, Pakistan took immediate steps to control the spread of the fatal disease.

Despite numerous social, cultural, religious, and political barriers, the Federal Government of Pakistan improved its level of preparedness by establishing centralized committees, such as the National Command and Operation Center (NCOC), to ensure a unified response to COVID-19 across the country [10, 11], but the NCOC predominantly comprised of federal ministers and the leading military leaders, outnumbering the provincial ministers [12]. This approach sidelined the provincial voices in decision-making, despite having crucial local knowledge about region-specific health needs and challenges [12]. The NCOC became a symbol of consolidated power, reflecting the power struggle between the federal and the provincial governments in Pakistan [12].

Pakistan experienced five waves of COVID-19 in two years. In response to these waves, the government imposed “smart lockdowns”, sealing off areas with positive cases of COVID-19. The government also utilised technology to raise public awareness, utilizing social media, print, and electronic media. The Government of Pakistan started its vaccine campaign approximately a year after its first reported case of COVID-19 (i.e., March 2021). The vaccination process involved registration using a computerised national identity card (CNIC) through an SMS, followed by vaccine administration at the nearest healthcare facility. The mechanism was progressively adapted to address emerging needs and incorporate lessons learned throughout the process. Vaccination cards were made compulsory at banks, hospitals, schools, offices, and other public places. However, refugee and migrant populations faced challenges in obtaining vaccination due to their ineligibility for CNICs, which were a prerequisite for registration. Eventually, in early July 2021, vaccination services were extended to refugees and migrants, who were allowed to register using passports or Proof of Registration (PoR) cards. Their information was documented separately in an Excel spreadsheet. Despite these efforts, Pakistan was unable to achieve the desired vaccination coverage due to various challenges such as vaccine hesitancy, supply chain issues, and logistical constraints.

Apart from these constraints, many people resisted vaccination. Existing vaccine hesitancy, particularly toward the polio vaccine, is rooted in misconceptions, myths, misinformation, and fears of side effects [13]. The failure of national immunization programs to eliminate polio from Pakistan led to complex barriers to vaccine acceptance, including lack of trust in healthcare systems, inequity, limited access to accurate health information, and cultural beliefs that can influence medical decision-making [14–16]. These existing concerns further complicated the uptake of COVID-19 vaccinations [14]. This hesitancy was further exacerbated by the emergence of viral variants with reduced vaccine efficacy and successive global waves of COVID-19, which undermined early hopes of controlling the pandemic through reduced transmission [13,17].

Pakistan faced additional challenges in addressing the ongoing health needs of approximately 2.4 million Afghan refugees, along with the risk of an influx of more refugees due to overtake of Taliban in Kabul [17, 18]. The COVID-19 pandemic exacerbated existing problems, as access to healthcare services became even more limited, leading to a deterioration in the health conditions of Afghan refugees in particular [17].

This context extends beyond local challenges, offering critical insights into global public health issues. Understanding the barriers to the access, utilisation, and impediments to COVID-19 vaccination among refugees, migrants in regular situations (MIRS), and migrants in irregular situations (MIIS) in Pakistan will provide a crucial lens for comprehending similar challenges in other resource-limited settings and among refugee and migrant populations worldwide. Considering this knowledge gap, Therefore, we aim to comprehend the perspectives of refugees and migrants regarding the COVID-19 vaccine. We seek to gain insights into their awareness levels and the challenges they encountered while accessing the COVID-19 vaccine in Pakistan. This study not only sheds light on the specific issues faced by this population but also offers valuable insights into the broader challenges associated with large-scale migration, thereby contributing to informed strategies for similar situations in the future.

## Objective

This study aimed to understand the behavioral and social drivers of the COVID-19 vaccine uptake and to identify the challenges and enablers affecting vaccine access among Pakistan’s refugee and migrant populations.

## Methodology

### Overview

This qualitative study was conducted in collaboration with the University of Geneva as part of a larger study conducted in six countries including Ecuador (Latin America), Nepal and Pakistan (South Asia), the Philippines (South-East Asia), Rwanda (Sub-Saharan Africa), and Tajikistan (Central Asia). In this paper, we report the findings from Pakistan only, following the Standards for Reporting Qualitative Research (SRQR) checklist (See S1 Checklist).

The study is grounded in the WHO Health Systems Framework, with its six system building blocks, and the Behavioural and Social Drivers (BeSD) of the COVID-19 Vaccination Framework [19, 20]. The six system building blocks are leadership and governance, healthcare financing, health workforce, medical products and technologies, information and research, and service delivery. The WHO Health System Framework not only informs the design and contents of our surveys but also helps enhance the actionability of our findings in terms of health system strengthening. The BeSD of the COVID-19 Vaccination Framework allows careful consideration of how individual, social, and structural determinants interact to impact willingness to vaccinate and uptake of vaccination.

### Setting

The study was conducted in Quetta (a city in the province of Balochistan), Karachi and Hyderabad (cities in Sindh province). These locations were purposively selected because of the high registered numbers of refugee and migrant populations. We were unable to interview refugees and migrants from Khyber Pakhtunkhwa despite the high number of Afghan refugees due to lack of permission and access.

### Participants

Participants included refugees, MIRS and MIIS (aged 18 years and above) who qualified for COVID-19 vaccination in Pakistan (Box 1). To ensure accurate identification of refugees, MIRS, and MIIS, we contacted the government district team which was further supported by the experience of the research team. Some participants self-reported their refugee and migration status at the time of enrollment. The study participants also included stakeholders (aged 18 years and above) such as refugee and migrant community leaders, government officials, development partners, representatives from NGOs, and health department officials from Sindh and Balochistan, who were responsible for overlooking the COVID-19 vaccination process, as well as civil society organizations within migrant and refugee communities.

**Definition of Refugee and Migrants (in regular and irregular situations)**

- *Refugee* – Individuals residing outside their country of origin due to fear of persecution, conflict, generalized violence, or other situations that have seriously disturbed public order and, as a result, require international protection.
- *Migrant* – Is a broad term which is not defined under international law. Migrants are people who move away from their place of usual residence, whether within a country or across an international border, temporarily or permanently, and for a variety of reasons. The term includes a number of well-defined legal categories of people, such as migrant workers; persons whose particular types of movements are legally defined, such as smuggled migrants; as well as those whose status or means of movement are not specifically defined under international law, such as international students.
  - *Regular migration* – Migration that occurs in compliance with the laws of the country of origin, transit, and destination.
  - *Irregular migration* – Movement of individuals that takes place outside the laws, regulations, or international agreements governing the entry into or exit from the State of origin, transit, or destination.

### Sampling

To capture varied perceptions, we adopted a non-probabilistic expert sampling strategy. This type of sampling approach involves selection of participants based on their knowledge, experience, or relevance to the research topic rather than using random sampling techniques. It is helpful in the early phases of a research project to gain in-depth insights into an issue, explore key themes and inform further avenues of inquiry [21]. We also adopted a snowballing sampling technique through personal contacts to identify relevant stakeholders and community members. We continued to interview participants until a level of saturation was achieved.

### Procedure

We conducted eight in-depth interviews (IDIs) with stakeholders and community leaders who were involved in the COVID-19 vaccination process. To ensure the voices of vulnerable populations were accurately represented, we conducted two focus-group discussions (FGDs) with five participants each, one with males and one with females, exclusively with community members including refugees and migrants in both regular and irregular situations residing in Pakistan. We used only FGD with community as this a sensitive community and were usually not available for individual interviews as they have their own fears and were more comfortable when discussing in groups.

The separation of these two groups in data collection helped maintain the integrity of each group’s perspectives. A defined number of participants were included, and there were no refusals or dropouts. No repeat interviews were conducted. Data collection activities/interviews in Karachi and Hyderabad were initiated on June 15^th^, 2022, and successfully concluded on June 30^th^, 2022, while, in Quetta, data collection commenced on June 20th, 2022, and concluded on July 15^th^, 2022.

We engaged a team of social scientists with relevant experience in qualitative methods and local languages. The interviews were conducted by trained researchers who had no relationship with the participants. The interviews were conducted in person, remotely via phone or videoconference, depending on the pandemic situation and the safety protocols in place. For in-person interviews, private locations were chosen to ensure confidentiality, including a community center for the FGDs, offices of stakeholders for IDIs, or other places of convenience for participants. For interviews through the videoconferencing platform, videos were kept off during the interviews to ensure the privacy of the participants. Consent was obtained from all the research participants before participation. For phone and videoconference interviews, verbal consent was obtained at the start of the interview, and participants were informed about the study’s purpose, confidentiality, and their right to withdraw at any time.

The research team conducted a relevant literature review on COVID-19 vaccination, refugee and migrant health, and barriers to healthcare access, followed by discussions within the team and incorporation of their input to finalize the probing questions. A semi-structured topic guide with open-ended prompts was designed for IDIs and FGDs, addressing key areas such as access, uptake, challenges and enablers in accessing COVID-19 vaccines by refugees and migrants in regular and irregular situations (See S1 Text). Feedback was incorporated from researchers with experience working in the community to ensure that the guide was culturally relevant and aligned with the study’s objectives. The guide was then pilot-tested on five individuals. Interviews with community members and leaders were conducted in the local language (i.e., Urdu), During the FGDs, the refugees and migrants were able to speak and understand basic Urdu language but we had support from Bengali and Afghan translators to ensure effective communication, particularly to address potential language barriers. Although translators were present as linguistic support, all participant responses were originally provided in Urdu language. This approach ensured accurate interpretation and comprehensive understanding of participants’ perspectives while maintaining the integrity of the data collection process. Interviews with other stakeholders were conducted in English or Urdu languages based on the preferences and language proficiency of the participants.

The interviews were audiotaped after obtaining consent from participants. All the interviews were de-identified and saved in the data repository using a unique identifier. All the identifiers were removed at the time of data transcription and translation. Bilingual research assistants (can speak English and Urdu language) with experience in transcription, transcribed and translated the audio records into English. Accuracy checks were done by comparing transcripts with the audio files. The IDIs on average lasted approximately an hour while FDGs on average lasted for an hour and a half.

### Analysis

All the de-identified translations were uploaded and analysed on QSR NVivo 12 software [22]. Thematic analysis was conducted using an inductive approach, where themes emerged directly from the data. Initially, a coding framework was developed by a team member based on a portion of the transcripts, using NVivo. The initial themes were reviewed and refined by the research team leaders. Two coders were involved in the analysis. The primary coder manually coded all relevant transcript data according to the identified themes, and this was reviewed and cross-checked by a secondary coder. Any discrepancies were resolved through discussion and consensus. Saturation was achieved when no new themes emerged during the analysis of additional interviews, indicating that the data collected was comprehensive and sufficiently represented the perspectives of the participants. The final themes were shared with all the team members, who reviewed, discussed, and agreed upon the final coding and theme categorization.

### Ethical Considerations and Maintaining Confidentiality

The study was reviewed and approved by the AKU Ethical Review Committee (ERC) and the National Bioethics Committee (NBC). Written consent was obtained from all the research participants. Individual names and personal information of respondents were kept confidential and personal identifiers were not used in any form of reporting. Subjects and respondents were free to stop interviews at any time or skip any questions they did not deem to answer. All data files were saved in password-protected files.

### Inclusivity in Global Research

Additional information regarding the ethical, cultural, and scientific considerations specific to inclusivity in global research is included in the Supporting Information (See S2 Checklist)

## Results

**Fig 1:**
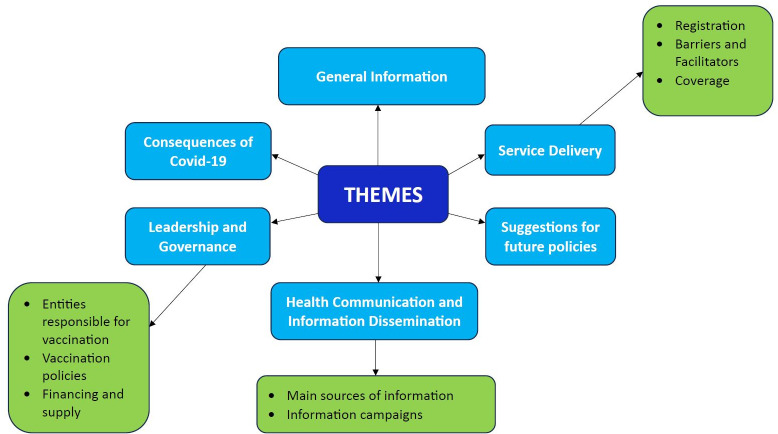
Themes emerged during the interviews with the refugee and migrant population.

A total of eight IDIs and two FGDs were conducted, comprising a total of 18 participants. Of the eight IDIs, two were conducted with community leaders from Bengali and Burmese refugee and migrant communities, while six were conducted with stakeholders, including government officials, development partners, and NGO representatives. The two FGDs, each with five participants, were conducted exclusively with refugee and migrant community members: one with Afghan males and the other with Bangladeshi and Burmese females. The characteristics of the study participants are presented in **Table 1**. The following major themes emerged from the interviews, which are also summarised in Fig 1.

**Table 1:** Characteristics of study participants.

Study participant characteristics (N: 18)	
	**n (%)**
***Age***	
18 to 29	2 (11.1)
30 to 49	14 (77.7)
50 to 64	2 (11.1)
***Gender***	
Male	13 (72.2)
Female	5 (27.7)
***Education***	
Primary	4 (22.2)
Secondary	3 (16.6)
Bachelors	1 (5.5)
Post-graduate	8 (44.4)
No education received	1 (5.5)
None of the above	1 (5.5)
***IDI participants (N:8)***	
Government officials	3 (37.5)
Refugee and migrant community leaders	2 (25.0)
Non-Governmental Organization	1 (12.5)
Development Partners	2 (25.0)
***FGD participants (N:10)*** ***Refugee and migrant communities***	
Afghanistan	5 (50.0)
Bangladesh	4 (40.0)
Burma	1 (10.0)

### Socio-demographics

The study findings revealed a diverse landscape of refugee and migrant populations in Pakistan. Participants identified several major migrant and refugee groups within the country. The findings also indicated that a large portion of refugees and migrants are settled in Karachi, Pakistan’s largest industrial hub, with many living in urban areas. It was noted that while almost half the population is fixed, the other half is mobile, constantly traversing between different cities. In terms of geographic distribution, Balochistan was highlighted as a key region hosting refugee camps, particularly in the districts of Quetta, Pishin, Loralai, Chaghi and Kila Abdullah. The Bengali communities predominantly comprised second and third-generation migrants and most were born in Pakistan, but still did not have any government-issued documentation and therefore had no access to many of the facilities and resources in the country.

Most of the interviewees knew of irregular migrants in Pakistan and were able to give largely varied estimations ranging from 20,000 to 500,000.

### Consequences of COVID-19

Most refugee and migrant community members and leaders discussed how livelihood was severely affected during the lockdown periods, leading to significant mental health strain that was accompanied by loss of income and sustenance. As the refugee and migrant population mainly comprised of daily wage earners, they faced a sharp decline in their earnings. Additionally, these participants described how a lack of identification documents prevented them from accessing basic services available to the general population, including food rations from support organizations and medical treatment at hospitals. Government hospitals were largely inaccessible, and although some private hospitals were willing to treat undocumented individuals, the cost was prohibitively high. Consequently, these communities found themselves trapped in a vicious cycle of hardship, struggling to access education, employment, and healthcare— all of which require some form of government-issued documentation. Amongst the migrant communities, the primary burden was not so much morbidity and mortality, but rather the loss of income and livelihood due to the pandemic.

*“Mostly migrants or refugees are daily wage workers and due to lockdown, they faced many challenges of getting food. Due to non-availability of identification documents, they couldn’t avail the facilities that the general population can.” – (IDI_NA_006)*

*“People who were previously able to afford eating three times a day were not even being able to manage one meal per day.” – (IDI_FI_005)*

*“Companies and bazaars, everything closed during lockdown so all the people in our community who worked here had no income. Everyone was affected, but no one died from Covid-19.” – (FGD_AA_009)*

### Leadership and Governance

#### Entities responsible for vaccination.

**Fig 2:**
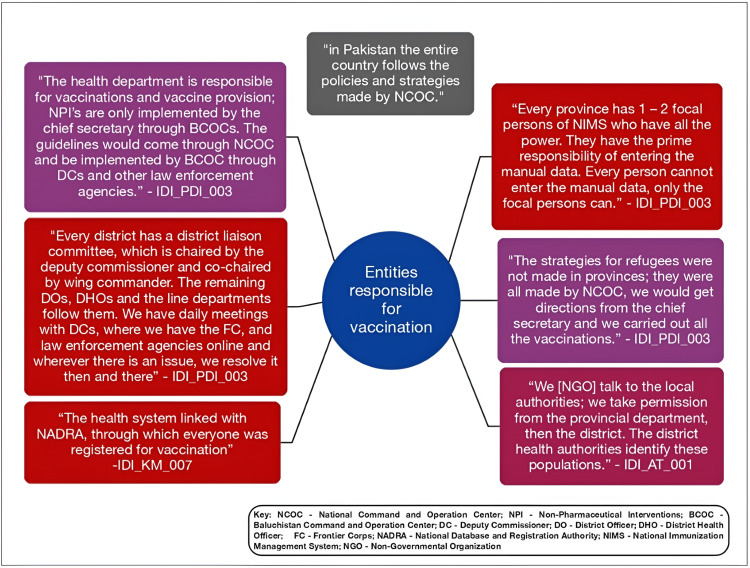
Entities responsible for the vaccination process in Pakistan.

At the government level, we found that the COVID-19 vaccination process throughout the country followed the policies and strategies developed by the National Command and Operation Center (NCOC). According to these guidelines, government officials reported that registration for vaccinations was done on national identification documentation (i.e., CNIC). *“During this outbreak for the first time in Pakistan’s history, the health system was linked with NADRA which is our national database, and through it everyone was vaccinated.” – (IDI_KM_007)* **Fig 2** details the various entities responsible for the vaccination process in Pakistan.

#### Exclusive vaccination policies.

Based on discussions with stakeholders, including those working with the government and with development partners, the COVID-19 vaccination process in Pakistan commenced in February 2021. The initial phase targeted healthcare providers followed by an age-based rollout starting with those aged 80 and above and gradually extending to schoolchildren.

*“To encourage vaccination, the government imposed strict regulations against entering banks, restaurants, malls, and other general public spaces for those who were not vaccinated.” – (IDI_PDI_003)*

A representative from Sindh said that some government employees in the province were bound to get vaccinated in order to get their salaries. Vaccination cards were made a mandatory requirement to access workplaces, schools, and hospitals for the general population.

#### Inclusive vaccination policies.

Government representatives and stakeholders from NGOs and health organizations discussed the absence of clear policies for the refugee and migrant population during the initial five to six months of the pandemic. Similarly, some refugee and migrant participants reported that, prior to June 2021, migrants who visited vaccination centers were completely refused by officials.

*“Our registration system, which by default only accepted NIC of permanent residents, in that specific condition, migrants and refugees who did not have an NIC were unable to register. They were in the mouth of the disease and very hard hit. There was no clear-cut policy for this population in the initial 5-6 months.” – (IDI_A_002)*

Later, vaccination policies for the displaced and refugee population were considered. For migrants in regular situations, government officials from Sindh and Balochistan explained that the policy declared that they would just need to show their passports, and their vaccination records would be manually recorded and maintained in Excel sheets by the districts. These records were then entered into the official database by the provincial Focal Person (FP). Similarly, the same strategy was employed for Afghan refugees and migrants who had a Proof of Registration (PoR) card, and vaccinations were carried out in refugee camps and migrant communities towards the end of 2021. The health department issued the vaccination cards and official stamping was done by law enforcement agencies.

*“In the fourth quarter of 2021, the government with the help of WHO formed a special policy for them and have started mobile vaccination teams for refugees and migrants, through district administrations. Special focus was placed on Afghanis” – (IDI_KM_007)*

When asked about the current vaccination campaign, a government official from Sindh province reported that they followed the policy of vaccinating everyone without discrimination based on documents. For unregistered persons, data was maintained manually, and they were issued simple vaccination cards as opposed to the vaccination cards given by the National Database and Registration Authority (NADRA) to registered persons.

“*Every vaccination center was given a simple proforma comprising of basic information like name, age, gender, and address of residence in English language for unregistered population. They were given simple manual vaccine cards, and they were told that they are not registered with NADRA which is why they can’t receive NADRA COVID-19 vaccine card, which every registered person gets.” – (IDI_KM_007)*

However, refugee and migrant participants expressed different views. A few community members shared that migrants in irregular situations and refugees who lacked any form of documentation were still not considered in the vaccination process. Although some government representatives were of the view that vaccinations were equally available to all groups, particularly in late 2021, many refugee and migrant community members reported that no special policies existed for them, mainly due to a lack of information dissemination and targeted awareness campaigns in their areas.

#### Financing and Supply.

Most government officials and health system representatives were of the opinion that there were no issues related to vaccination supply in healthcare facilities or communities across Pakistan. They emphasized that COVID-19 vaccines and vaccination services were provided free of cost to everyone in the country, whether they are Pakistani nationals, refugees, or regular or irregular migrants amongst those who were eligible for vaccination. When asked whether they received special directions from NCOC regarding vaccinating refugees, a government official shared:

*“Free of cost, all the services they received were free. The same services that were provided to Pakistani nationals were provided to the Afghan refugees.” – (IDI_PDI_003)*

Within the communities of migrants and refugees, none of the participanted reported being charged for vaccination. However, some community leaders shared incidents of fraudulent practices involving vaccination cards. They reported instances where vaccination cards were being made for a certain sum of money, without having administered the vaccine. This was reportedly done to satisfy vaccine requirements without having to undergo vaccination—potentially appealing to vaccine-hesitant individuals. When asked if people paid for the vaccine, a community representative from an Afghan migrant community responded:

*“Yes, not to get vaccinated but to make a vaccination card.” – (FDG_AA_009)*

Some community members further elaborated that approximately 10% of the people in that community paid between 200 to 3000 Pakistani Rupees to obtain a card without getting vaccinated. A community member indicated that health workers facilitated these schemes, exploiting the lack of proper documentation and perpetuating mistrust in vaccination campaigns.

### Health Communication and Information Dissemination

#### Information campaigns.

Information related to COVID-19 was disseminated via mass media campaigns and awareness programs. Print media was utilized more than electronic media due to the lack of resources. A health department representative discussed the process, whereby the health education section produced and distributed the information, education, and communication (IEC) material provided by the United Nations International Children’s Emergency Fund (UNICEF). This information was translated mostly into Urdu, or the local language of the area most commonly *Pashtu*. Banners were also displayed on the vehicles utilized by mobile vaccination teams, and microphones were used to spread messages on the streets in some localities to ensure greater awareness.

Most communities relied on newspapers, radio, and television broadcasts, and community networks for information related to COVID-19 vaccination. Only a few community members reported accessing local authorities’ statements. In our discussions, the opinions of migrants and refugees differed from government representatives on public health campaigns specifically targeting migrants and refugees. Most refugee and migrant community members and leaders said that media efforts by NCOC to spread information did not reach most of the targeted communities. When asked about specific campaigns in their community, a community member indicated lack of exposure to these efforts:

*“We do see the cars, we hear of them, but we don’t know which organizations send them” – (FDG_CW_008)*

Some stakeholders, including a representative working with a development partner, discussed the lack of special promotional campaigns within refugee camps in Balochistan. They shared that health workers went house-to-house, to ensure everyone in the area was vaccinated. Many NGOs were involved in the vaccination process and collaborated with migrant and refugee communities and religious leaders, obtaining permission and guidance to run the vaccination process specifically for the community.

*“We have these mobile vans, and mobilizers, who go house to house, they take permission from a male if a male is available, then start talking to the females. They tell them a bit about COVID-19 and the vaccine, why it is important and basically communicate vaccine safety information.” – (IDI_AT_001)*

Despite the government’s announcement of door-to-door COVID-19 vaccination services, many participants from Bengali migrant communities complained of a lack of mobile vaccination teams and inefficiency in door-to-door visits. Ineffective communication and persuasion were issues in Afghan migrant communities. When asked about campaigns in their communities, refugee and migrant community members almost unanimously disregarded these efforts due to their religious leaders speaking against vaccinations:

*“We asked the religious Imams [leaders], why should we not get vaccinated. They say a person who lives in a very neat and clean environment, when such a virus infects them they become ill. People like us have so many germs that they don’t allow Corona to enter our bodies. Our Islamic leaders tell us, the vaccine can result in death after two years”*

While most community members were unsure about the vaccination coverage among the refugee and migrant populations, they reported it to be approximately 21 to 40% in their communities, indicating low vaccination uptake. When asked about the effectiveness of mobile campaigns in their area, some participants expressed dissatisfaction with the efforts. For instance, an Afghan refugee community member remarked:

*“Many people [mobilizers] come, they visit the union, sit around for a while, have tea and leave.” – FDG_AA_009*

This highlights the perceived inadequacy of mobilization efforts, with participants implying that the presence of mobilizers did not necessarily translate into effective vaccination outreach or significant coverage improvements.

### Service Delivery

#### Registration.

We asked participants how registration for vaccination was carried out. The National Database and Registration Authority (NADRA) introduced the National Immunization Management System (NIMS) according to which eligible persons would send a message containing their national identity card number to a local toll number, i.e., 1166 to prompt a confirmatory message containing a code and location of their vaccination centre. Some participants, both community members and government officials, discussed the complexity and flaws in this system; it was possible to experience a delay of up to 10 days in receiving the confirmatory message. Another problem was that if persons registered in a specific city were currently residing in another city, they would receive the location of a vaccination centre in the city where their national identity card was originally registered. For such people who were unable to travel and ended up getting vaccinated at a different centre, there arose the inevitable problem of data management.

As mentioned above, a large majority of the refugee and migrant population did not possess any identification documents issued by the government. As a result, many participants said they were unable to register via the introduced method for at least the first five to six months of the vaccination process. We were told that towards the end of May and early June 2021, the National Command and Operation Center (NCOC) relaxed the requirement of prior registration and appointment for vaccination and people were asked to report to any vaccination centre and get vaccinated. The requirement for national identity card was also relaxed and manual entry of data was allowed for those who did not have documentation. Some Afghan refugees and migrants were issued a local registration card known as the PoR card, while some had an Afghan passport known as *Tazkira*. It was announced that vaccinations would be done for everyone, including those possessing the aforementioned documents and even refugees and migrants who did not have any documentation. Since the registration system did not facilitate the entry of any data other than national identity card, participants said that registration was entered manually and maintained by districts. **Table 2** details the views of migrant community members and government representatives regarding the barriers and facilitators of accessing the COVID-19 vaccine.

**Table 2:** Barriers and facilitators of accessing COVID-19 vaccine.

	BeSD Framework Component	Issues for Migrants and Refugees	Description	Quotations
**Barriers**	Thinking and Feeling	Lack of awareness and education Perceived disease risk	In certain communities, resistance to COVID-19 vaccination stemmed from a lack of awareness about the disease and its prevention. Many individuals did not understand the effectiveness of the vaccine in preventing severe illness. This knowledge gap was compounded by the absence of widespread education and awareness campaigns in some areas. Participants noted that door-to-door vaccination drives, which could have increased outreach, were also missing in these communities. A significant barrier to vaccination was the denial of COVID-19’s existence. This perception was particularly prevalent among Pashto-speaking populations and others, where the disease was dismissed as a Western propaganda. As one participant stated, “If one does not accept the reality of the disease, how would he get vaccinated?” Another added that this denial was fueled by a lack of clarity about COVID-19, particularly among the undereducated, who questioned its legitimacy altogether.	*“They mostly do not have the knowledge of how effective the vaccine is in prevention.” – IDI_A_002* *“There is some resistance to the vaccine, firstly because people are not clear about COVID-19 itself. Some people say COVID-19 is nothing. Then, there is undereducated population, I wouldn’t say illiterate, but the problem is that they question the reality of COVID.” – IDI_AT_001* *“An important thing to note is that the Pashtu speaking people here do not believe in Corona. If one does not accept the reality of the disease, how would he get vaccinated” – FDG_AA_009*
	Social Processes	Fear of identification by authorities Fear of side effects Gender and cultural norms	Fear of being identified by authorities was a significant barrier among unregistered populations, such as refugees and migrants. Even though only basic information like name, age, and address was collected during manual data entry, many feared being tracked and potentially deported if their home country’s situation stabilized. Concerns about vaccine side effects were also prevalent, exacerbated by religious leaders spreading misinformation. Common rumours included severe consequences such as death within two years, infertility, and other health complications. These fears were heightened by the unregistered population’s limited access to public healthcare services. Gender and cultural norms presented additional barriers, particularly among Afghan refugees. Social practices like pardah restricted women from leaving their homes or interacting with male vaccinators. As a result, vaccination efforts often had to be conducted door-to-door for women, and even then, male health workers were sometimes refused. This, coupled with a lack of female vaccinators, further decreased vaccine uptake.	*“Majority of this volatile refugee population is unregistered, as they have no records. There was always a fear that if they register and were identified, in the next 2-3 years if the situation in their home country improves they would be sent back. The next major barrier would be fear of side effects of vaccination.” – IDI_A_002* *“Also, a big issue was fear of side effects, that if something were to happen to them where would they go to seek care since they can’t seek treatment from public sector facilities” – IDI_KM_007* *“Our Islamic leaders tell us the vaccine can result in death after two years” – FDG_AA_009* *“There are a few challenges, with females for example, we must go to their house and vaccinate them. They are not going to go out of their house, the “chaar deewari”. They also sometimes refuse to get vaccinated by males.” – IDI_AT_001*
	Practical Issues	Accessibility Documentation challenges Falsification of vaccination records Language barriers	In many communities, there was a strong willingness among the unregistered population to get vaccinated, but practical barriers prevented access. Some participants shared how individuals in their communities faced numerous obstacles in obtaining vaccination due to documentation issues. For example, when migrant workers lacked proper documentation, such as a CNIC, they were often unable to receive vaccinations. In some cases, health workers marked them as vaccinated on forms without administering the vaccine. This practice eroded trust in the vaccination system, with one participant describing how health workers simply ticked the box, leaving those without documentation unvaccinated but falsely reported as vaccinated. Another issue that surfaced was the purchase of fake vaccination cards, where some people paid money for proof of vaccination but did not receive the actual vaccine, which contributed to a sense of complacency. As one individual explained, after obtaining a fake card, there was no perceived need to get vaccinated. Access to vaccination centers was also a significant challenge. Initially, only a few vaccination centers were available, leading to long lines and long wait times. Those with work obligations found it especially difficult to dedicate hours to waiting in line. While language barriers were generally not widespread, they were a concern for some populations, particularly refugees and migrants. For example, in a district with a large Hazara community, language differences caused communication issues. The Hazara community primarily spoke Persian (Farsi), but the vaccination teams were often staffed by Pashto speakers, creating confusion and limiting the effectiveness of information dissemination. One participant observed, “How will the six women who cannot understand Pashto be supported?” highlighting the gap in communication. This barrier reinforced the need for multilingual outreach and educational materials in diverse areas.	*“Accessibility was also a problem. Initially, only select 3-4 centers were offering vaccinations and naturally there would be a lot of rush. Those who would be out to work early morning, it would be a problem as they were unable to afford waiting in line for 3 or more hours to get vaccinated.” – IDI_A_002* *“Thirdly some people who paid money to obtain fake vaccination cards, they didn’t feel the need to get the vaccine since they had already got proof of vaccination” – IDI_NA_006* *“We don’t have CNIC, so they [health workers] don’t give us the vaccine and just put a tick on their forms. They come and say okay don’t get vaccinated, no problem, we are going to tick yes on the form regardless. Who is going to go and investigate this issue and keep tabs on it? They are playing with the sentiments of the poor.” – FDG_CW_008* *“The first thing I noticed was that the Hazara people were speaking in their Persian (Farsi) language, and there was not a single Persian-speaking individual in our team. Just imagine. I told the coordinator, you are Pashtu speaking, tell me how many people here speak Pashtu. He said many do. I randomly asked ten women if they can speak Pashtu, four could, six could not. I said what will be done about the six who could not understand Pashtu?” – IDI_AT_001*
**Facilitators**	Social Processes	Trust building through provision of medicines	An effective strategy to overcome vaccine resistance was the provision of basic medicines for common illnesses alongside COVID-19 vaccinations. Initially, significant resistance was observed, particularly among migrant and refugee populations. However, this approach helped address immediate health needs while building trust in the healthcare system. This strategy not only strengthened the bond between health workers and communities but also encouraged a positive attitude toward vaccination, contributing to vaccine uptake.	*“During October 2021, in the beginning of vaccine camps we faced a lot of resistance from them but when they were facilitated with some basic medicines for different diseases, which created a bond of trust, they were coming happily to vaccine centers seeking COVID-19 vaccination.” – IDI_KM_007*
	Motivation	School requirement	Mandatory school vaccination requirements served as a strong motivator for previously hesitant individuals. Many community participants reported that parents who were initially unwilling to get vaccinated became eager to comply to ensure their children could attend school without issue.	*“Now it is a little easier, since children are going to school it is the school’s requirement to get vaccinated and so they do. Door to door vaccinations are also happening, however, still those who don’t have CNIC aren’t getting vaccinated” – IDI_CW_008*
	Practical Issues	Door-to-door vaccinations Availability and ease of access: Organizations facilitating vaccination camps Border vaccination camps Respect form healthcare workers	To address low vaccination uptake among women and underserved populations, female mobilizers were deployed to conduct door-to-door vaccinations. These efforts included data collectors proficient in local languages to ensure inclusivity, even for those lacking official documentation. A four-pronged strategy involving fixed sites, mobile teams, health house taps, and social mobilizers was instrumental in reaching refugee and migrant communities. NGOs played a critical role in facilitating vaccinations, particularly in regions like Balochistan. Vaccination teams and camps were set up at the Chaman border with Afghanistan and the Taftan border with Iran to vaccinate migrants and refugees immediately upon entry. Special camps were also established for Iranian refugees in Hazara town, Quetta. In the latter part of 2021, the government, supported by WHO and UNICEF, focused on setting up dedicated vaccination camps in Karachi for refugees and migrants. Migrants with documentation experienced smooth and respectful processes at designated centers, where healthcare workers maintained a welcoming and respectful attitude. This approach fostered trust and created a positive atmosphere, encouraging others in their communities to get vaccinated. These combined efforts increased vaccination uptake in previously hesitant populations.	*“During October 2021, in the beginning of vaccine camps we faced a lot of resistance from them but when they were facilitated with some basic medicines for different diseases, which created a bond of trust, they were coming happily to vaccine centers seeking COVID-19 vaccination.” – IDI_KM_007* *“The aim of this project is basically to vaccinate all the refugee population. Also, the second objective is to increase the uptake of COVID 19 vaccines in populations where the uptake is low. So, because the vaccine uptake was low in Quetta, that’s why the project is taking place there, for the Afghan population there as well.” – IDI_AT_001* *“Yes, the general attitude was good, the vaccination process was smooth and comfortable, and there was no stress. No extra questions were asked of us.” – FDG_AA_009* *“Vaccinations are being done for everyone. There are vaccination teams on the Chaman border (Afghanistan), Taftan border (with Iran) etc. where vaccinations are being carried out” – IDI_PDI_003*

Acronyms: CNIC, computerized national identity card; NIMS, national immunization management system; NCOC, national command and operation centre; NGO, non-governmental organizations.

*“In 2014 my CNIC (national identity card) expired, I reapplied. I finally got my CNIC in 2019; you cannot imagine how many troubles I had to face.” -FDG_CW_008*

#### Coverage.

A large variation in statistics regarding vaccination coverage was observed between government representatives and local refugee and migrant communities. Within an Afghan migrant locality in Karachi, participants reported approximately 80% of the people in their community are unvaccinated to date mostly due to lack of awareness regarding the disease and the benefits of vaccination, misinformation, and fear of side effects. Similarly, in discussions with Bengali migrants, participants shared that only half of their community was vaccinated while the other half was not, largely due to lack of documentation, despite the new directives by NCOC. Government representatives and stakeholders involved in the vaccination process suggested that majority of refugees, regular migrants, and irregular migrants have been vaccinated, although this estimate was based on official records and general knowledge.

*“According to the best of knowledge 50% to 60% of the unregistered population of refugees and migrants have been vaccinated and the remaining 40% is due to their unwillingness.” – IDI_KM_007*

These figures are qualitative estimates based on participants’ perceptions and experiences, rather than precise quantitative data. Government representatives provided their estimates based on their knowledge of vaccination campaigns, while community members’ views were shaped by personal observations and interactions within their localities. As such, there is a degree of uncertainty and variation in the perceptions of vaccination coverage, reflecting differing experiences between stakeholders and community members. The estimates presented should be considered as informed approximations rather than exact figures.

### Suggestions for Future Policies

Participants highlighted several actionable steps to improve vaccination access for refugee and migrant populations, emphasizing the need to address critical barriers and build on existing facilitators. Given that lack of documentation emerged as the most cited barrier, addressing this was identified as a top priority by participants. Providing documentation for refugee and migrant populations was viewed as a foundational step to improving access to essential health services, including vaccination. As one community representative from a migrant community highlighted, *“Many of us were born here, yet we still face the same challenges as those who have recently arrived.”* Providing documentation, participants discussed, could strengthen the sense of nationality and belonging, fostering a more inclusive environment for these communities. Specific recommendations included creating temporary identification cards or introducing alternative registration mechanisms for undocumented individuals to ensure immediate access to vaccination without delays.

A recurring suggestion was the need to simplify the registration process for vaccinations. Several participants, including government officials and health system representatives identified the registration process as a major hurdle for vulnerable populations who lacked necessary documentation. Participants suggested removing requirements such as Computerized National Identity Cards (CNICs) or proof of residence to facilitate easier access. As one official stated, *“The aim should be to streamline the process to prevent delays, curb the rapid spread of disease and ensure that unregistered individuals are not overlooked.”.*

A key recommendation from refugee and migrant community members and leaders was the need for culturally and linguistically appropriate communication strategies. They suggested that vaccination campaigns target communal spaces like mosques, community centers, and schools in addition to door-to-door outreach. Participants further recommended engaging trusted local influencers, including religious leaders and community representatives, to combat misinformation, and vaccine hesitancy and build trust. Strengthening vaccine safety communication was also identified as a priority, with participants describing this as a weak aspect of current campaigns.

Health department officials suggested offering comprehensive healthcare packages that include vaccines along with other essential health supplies to encourage higher vaccine uptake. Both community leaders and health officials emphasized the importance of collaboration between refugee organizations, such as UNHCR, and the health sector to ensure the needs of both registered and unregistered migrants and refugees are addressed in policymaking. Participants also suggested involving local law enforcement agencies in identifying and supporting vulnerable populations during vaccination drives.

By incorporating these participant-driven recommendations, addressing documentation barriers, simplifying registration processes, enhancing communication efforts, and fostering multi-sectoral collaboration, policymakers can improve vaccine access and uptake among refugee and migrant populations in Pakistan, fostering a more equitable and inclusive public health response.

## Discussion

This qualitative study aids in understanding the behavioural, social, structural, and procedural factors affecting the COVID-19 vaccine and also in understanding the context of low vaccination rates among refugees and migrant populations residing in Pakistan. In this qualitative study majority of the respondents were daily wage earners who were among the most compromised populations at the time of the COVID-19 outbreak. Participants faced hardships during COVID-19, which included loss of jobs, drop in salaries, and difficulty in accessing healthcare due to the unavailability of valid identification documents. Amidst COVID-19, the NCOC committee was formed to play a central role in coordinating the national response and developing policies to improve the vaccination coverage, however, no such policy was passed for refugees and migrant populations in the initial three to six months of the outbreak. Participants including stakeholders working with the government and development partners in related discussions reported that the early phases of the vaccination drive were primarily focused on individuals with CNICs, making it inaccessible for the migrants and refugees. Additionally, provinces were not extensively involved in the formulation of vaccination policies, potentially limiting localised strategies that could have addressed the unique needs and challenges of the community [12].

According to the stakeholders working with the government and with development partners, vaccination campaigning in Pakistan was initiated in February 2021 for the public, but there was a lag of approximately five to six months before it was made accessible for the migrants [23, 24]. Later vaccination was provided to refugees and migrants by munaul registrations on an Excel sheet. Despite the willingness, refugees and migrants reported not being vaccinated due to a lack of formal documents leading to restricted access to healthcare, education, and other necessities.

Misconceptions and vaccine hesitancy also prevailed within these communities. Some participants were sceptical about getting vaccinated and few considered it as a foreign conspiracy or feared the vaccine could trigger infertility [25, 26]. Female respondents mainly reported on cultural barriers including pardah, unavailability of female vaccinators, and permission issues to leave home alone. While others had concerns about being deported [25, 26], while some feared side effects, disease risk, and death due to rumours spread by religious leaders. Most of the government officials and health system representatives reported no issues in terms of financing and supply for vaccination, while respondents including government officials, health system representatives, refugees and migrants also reported on the provision of free vaccination for all, however many refugees and migrants complained of being charged for creating fake vaccination cards without receiving the actual COVID-19 vaccine. The act of provision of fraudulent cards by healthcare workers appealed to vaccine-hesitant members of the community but it also led to mistrust of vaccines among people. Cases of counterfeit COVID-19 vaccination worth a million dollars and provision of fake vaccination were reported in countries like India, Nigeria, South Africa, Mexico, and the United States of America were reported irrespective of immigration status [27–30].

The stakeholders reported on introducing door-to-door and mobile vaccination campaigns to improve coverage. Participants reported on the lack of campaigning programs by NGOs and the government, particularly in refugee camps of Balochistan. These issues also persisted in different countries irrespective of the type of vaccine, reflecting systemic challenges in vaccination efforts [31, 32]. Studies have also reported on lack of trust, awareness, and disbelief in vaccines among the Pakistani population [33, 34]. A global analysis from 113 countries reported a positive relationship between vaccine willingness and trust in government and a negative link to distrust in government [35].

Despite the efforts to maximise the COVID-19 vaccine coverage by the federal government, a large proportion of refugees and migrants remain unvaccinated. The low vaccination rates within the targeted population could be due to limited access to information or a lack of vaccine promotion in refugee camps. Print media was utilised more than electronic media for vaccine campaigns due to the lack of resources, however, in several other countries social media was considered a powerful and equitable source of all the information related to the COVID-19 vaccine. Similar to our study finding, studies involving migrants and refugees from other countries including developed countries reported limited access to information due to the unavailability of information in their native languages [36]. Some countries like Germany, Denmark and Canada introduced multi-lingual websites to facilitate communications regarding COVID-19 vaccines [37, 38].

The COVID-19 pandemic worsened the disparities between countries with low income and those with high income, with a greater impact on the poorer and vulnerable populations including refugees and migrants [36]. The International Organization for Migration (IOM) reported on the inclusion of only regular migrants and refugees in COVID-19 vaccine campaigns, with very scanty evidence on the inclusion of irregular migrants across the world [36,39]. In Peru, irregular migrants were not included in the COVID-19 vaccination plan [40], in Ecuador, regular migrants were included in the third phase of vaccine campaigning [41], in Brazil vaccinations were provided to all irrespective of their migration status but irregular migrants were not considered a priority and did not receive any clear instruction on accessing the COVID-19 vaccine [36,42]. Meanwhile in Chile, the ministry excluded people on transition visas from the vaccination plan and the guidelines did not contain any information on the migrant population [36]. Few countries did take steps to accommodate refugees and asylum seekers during the pandemic. For example, the government of Portugal temporarily extended full citizenship rights to all migrants and asylum seekers, eliminating barriers to healthcare access, while the Home Office in England provided accommodation to thousands of previously refused asylum seekers during the start of the outbreak. Germany also supported refugees during the pandemic while many countries continued with deportation amid travel restrictions.

Apart from disparity in guidelines and access to vaccines, studies have shed light on numerous other challenges faced by migrants and refugees during the pandemic. Factors like pre-existing health conditions, poverty, crowded work and living conditions, high-risk jobs, fewer job opportunities, and limited access to healthcare contributed to high vulnerability to infections and other morbidities [36,43,44]. Compounding these challenges, the ‘covidisation’ of healthcare has led to deprioritisation of non-COVID-19 services, including crucial services like sexual and reproductive health and mental health [45–47]. These multifaceted issues have made migrant and refugee populations more susceptible to infections and mortalities [47]. The COVID-19 crisis has highlighted the critical importance of understanding and engaging the health of migrant populations which has been neglected in the global health security and preparedness plans amidst past epidemics [47–49].

### Limitations of the Study

The study has several limitations that should be acknowledged. The findings are not generalizable to high-income countries as it focus on refugees and migrants residing in Pakistan only. We were unable to reach and capture views of refugees and migrants from all the provinces of Pakistan. Thus, given the limited geographic scope of the study, the findings may not represent all the refugees and migrants residing in Pakistan. The study focuses on a specific population (i.e., refugees and migrants) of Pakistan limiting the generalization of findings to the country’s diverse and heterogeneous communities. The small sample size is another limitation, inherited in qualitative studies. The small sample size of the study limits the generalizability of the findings, making it difficult to draw broader conclusions for Pakistan. However, it does not compromise the validity of the study, as our sample was sufficient to reach data saturation.

The nature of the study may have also introduced recall or reporting bias. This was overcome by cross-checking among the KIs and through available literature on the web. People might have been apprehensive in replying as they would have fears because of their status, and some being unregistered.

## Conclusion and Implications

The findings of this study highlight the need for targeted strategies to improve vaccination access among refugee and migrant populations. A key recommendation is the provision of identification documentation to refugee and migrant populations on a priority basis, not just for emergency situations but for daily life and equal access to facilities. Additionally, the health registration and access mechanism should be simplified especially in emergency situations such as the COVID-19 pandemic for migrants and refugees and these populations should be given a separate focus in all policies especially during emergency situations.

While the study’s findings are specific to Pakistan, it offers valuable insights into the broader global context of vaccination challenges among refugees and migrants. The current evidence shows a dramatic decline in routine vaccination rates globally [50], aggravating the risk of vaccine-preventable diseases while making this research particularly relevant. The study suggests that healthcare systems must prioritize accessibility, reduce administrative complexities, and develop targeted approaches that recognize the unique challenges faced by migrant and refugee populations. By contextualizing these findings within the current global public health scenario, the study provides crucial guidance for developing more inclusive and effective vaccination strategies that can be adapted across different regional contexts.

The research underscores the critical need for policymakers to collaborate and actively engage with refugee/migrant-focused agencies and community representatives, as these individuals and organisations are crucial in identifying and reaching out to marginalized and vulnerable population groups. This collaboration will help in addressing systemic barriers and developing targeted population and equitable approaches to access and improve the health of vulnerable populations.

## Supporting information

S1 Checklist Standards for Reporting Qualitative Research (SRQR). (PDF)

S2 Checklist Inclusivity in global research. (PDF)

S1 Text A semi-structured guide for in-depth interviews. (PDF)
